# Shared molecular features and potential diagnostic biomarkers between ulcerative colitis and sarcopenia: an integrative bioinformatics analysis with preliminary experimental validation

**DOI:** 10.3389/fimmu.2026.1801914

**Published:** 2026-04-15

**Authors:** Beiying Deng, Yinghui Liu, Pengzhan He, Nian Wang, Lin Xu, Jingjing Ma

**Affiliations:** Department of Geriatric, Renmin Hospital of Wuhan University, Wuhan, Hubei, China

**Keywords:** biomark, fatty acid metabolism, machine learning, sarcopenia, ulcerative colitis

## Abstract

Sarcopenia and ulcerative colitis (UC) may share potential biological links through mechanisms involving immune dysregulation, gut microbiota imbalance, and inflammatory pathway activation. This study aimed to identify fatty acid metabolism-related shared genes between UC and sarcopenia. A total of 277 shared differentially expressed genes (sDEGs) were identified, which were mainly enriched in immune-, inflammation-, and metabolism-related pathways. By intersecting the sDEGs with fatty acid metabolism-related gene sets, 13 fatty acid metabolism-related shared differentially expressed genes (FAM-sDEGs) were obtained, and unsupervised clustering based on these genes stratified UC patients into distinct molecular subtypes. Using machine learning and feature-ranking methods, PHYH and HSD17B3 were ultimately identified as key hub genes. ROC analysis showed that these two genes exhibited diagnostic value for both sarcopenia and UC across multiple datasets. In addition, the expression of the hub genes was significantly associated with immune cell infiltration profiles and was further validated in independent datasets. Preliminary validation in a UC cell model confirmed the downregulation of PHYH, while immunohistochemical analysis of colonic tissues further supported the downregulated expression of PHYH in patients with UC, consistent with the bioinformatics results. Collectively, these findings suggest that fatty acid metabolism may play an important role in the shared molecular features between UC and sarcopenia, and that PHYH may serve as potential diagnostic biomarkers.

## Introduction

Ulcerative colitis (UC) is a chronic, relapsing inflammatory disorder of the colonic mucosa. Its clinical course and treatment response vary widely across patients, reflecting substantial molecular heterogeneity (1). Although immune-targeted therapies have advanced, robust molecular stratification and broadly applicable diagnostic or risk-assessment biomarkers remain limited (2). A clearer map of the gene networks that shape UC—and tools that translate those signals into clinically useful predictions—would support more precise patient management (3).

Sarcopenia, characterized by progressive loss of skeletal muscle mass and function, is frequently accompanied by persistent inflammation, nutritional imbalance, and metabolic disruption (4). UC may predispose individuals to sarcopenia through long-standing inflammatory burden, altered nutrient absorption, and systemic metabolic remodeling. Conversely, changes in muscle metabolism and immune tone may influence intestinal inflammation and disease trajectory (5, 6). Although some clinical studies have suggested that patients with UC may be more prone to developing sarcopenia (7–9), the current evidence is largely derived from observational studies with limited sample sizes, and there is considerable heterogeneity in study populations and diagnostic criteria. Therefore, it is still premature to conclude that a clear and well-established clinical association between the two conditions has been definitively confirmed. Rather than directly emphasizing a clinical association, it may be more appropriate to investigate the potential molecular links between UC and sarcopenia from the perspective of shared inflammatory, metabolic, and immune regulatory mechanisms.

Fatty acid metabolism lies at the intersection of energy homeostasis and immune regulation and may represent an important entry point for understanding the shared pathological basis of UC and sarcopenia (10, 11). Previous studies have shown that, in UC, mucosal inflammation is often accompanied by marked metabolic reprogramming, particularly impaired fatty acid oxidation and abnormalities in mitochondrial and peroxisomal function (12); these alterations can further disrupt epithelial energy supply, redox homeostasis, and barrier integrity, thereby promoting persistent inflammatory responses and mucosal injury (13, 14). In parallel, reduced fatty acid oxidation, mitochondrial dysfunction, and energy stress in skeletal muscle are considered key metabolic features in the development and progression of sarcopenia and frequently occur in the setting of chronic inflammation (15, 16). Beyond its role in energy metabolism, dysregulated fatty acid metabolism may also influence the generation of lipid mediators, especially arachidonic acid-derived metabolites and other eicosanoids, thereby modulating immune cell recruitment, inflammatory polarization, Th17-related pathways, and cell adhesion and trafficking programs, which highlights the close interplay between fatty acid metabolism and the immune microenvironment (17). Therefore, investigating the “fatty acid metabolism-immune microenvironment” axis may help elucidate the shared molecular basis linking UC and sarcopenia (18, 19). Consistent with this concept, our enrichment analysis of shared differentially expressed genes revealed significant involvement of lipid- and metabolism-related pathways, particularly arachidonic acid metabolism, which further supported our focus on fatty acid metabolism-related genes for subsequent intersection analysis, UC subtyping, and biomarker identification.

In this study, we integrated publicly available transcriptomic datasets to identify shared differentially expressed genes (sDEGs) between UC and sarcopenia, and used GO/KEGG enrichment to delineate key pathways. We then intersected sDEGs with fatty acid metabolism gene sets to derive FAM-sDEGs and evaluated their functional links using a protein–protein interaction (PPI) network. In the UC cohort, unsupervised clustering based on FAM-sDEGs was performed to define metabolism-related subtypes, and GSVA/ssGSEA were applied to characterize pathway differences and immune infiltration patterns. Next, we applied LASSO, SVM-RFE, and random forest to prioritize hub genes, with focused validation of PHYH and HSD17B3 in the training and external cohorts, and constructed a logistic regression–based risk model to assess its discriminatory performance for UC and its generalizability to sarcopenia-related samples.

## Methods

### Data sources

**Figure 1:**
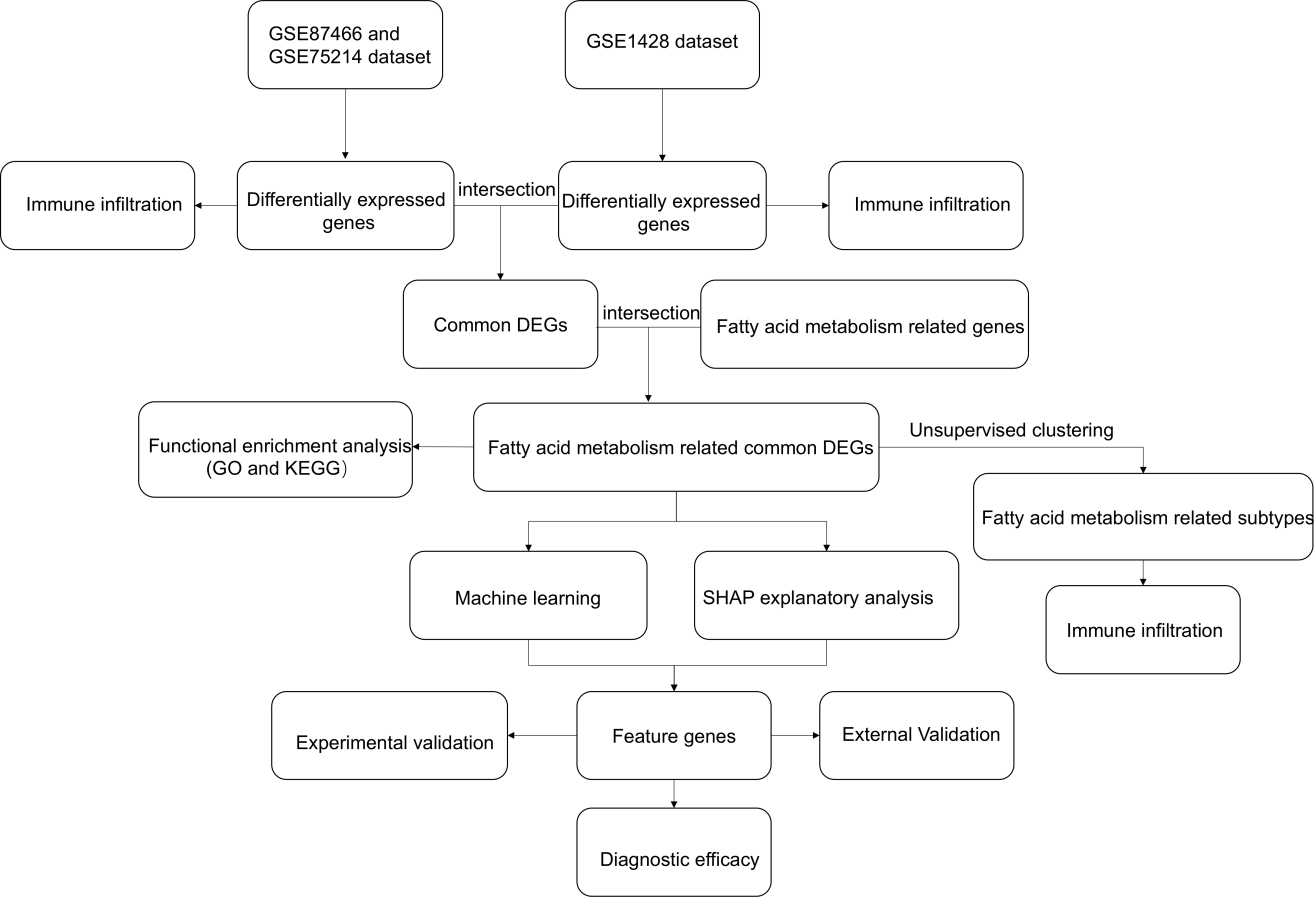
Flowchart of the study. GO, Gene Ontology; KEGG, Kyoto Encyclopedia of Genes and Genomes; DEGs: Differentially Expressed Genes.

Gene expression data were obtained from the Gene Expression Omnibus, selecting only human samples using the keywords “ulcerative colitis” and “sarcopenia.” GSE87466 (GPL13158) included 87 UC colonic mucosa and 21 controls, GSE75214 (GPL6244) contained 74 active UC samples and 11 controls, GSE206285 (GPL13158) comprised 550 UC samples and 18 controls, and GSE1428 (GPL96) consisted of 12 sarcopenic and 10 healthy skeletal muscle specimens. Genes associated with fatty acid metabolism were compiled from prior literature (**Supplementary Table 1**). The overall workflow of the study is summarized in **Figure 1**.

### Identification of differentially expressed genes

The UC datasets GSE87466 and GSE75214 were first merged, and batch effects were corrected using the ComBat function in the R package “sva”. Differentially expressed genes (DEGs) were then identified from the batch-corrected combined UC dataset and the sarcopenia dataset GSE1428 using the R package “limma”. DEGs were defined as genes with |log_2_FC| > 0.3 and adjusted P < 0.05 in the UC dataset, and |log_2_FC| > 0.3 and P < 0.05 in the sarcopenia dataset. Shared genes potentially contributing to both UC and sarcopenia were identified via Venn diagram analysis, followed by functional enrichment assessment.

### Machine learning algorithms

Shared DEGs between UC and sarcopenia were further refined using three established machine learning approaches: LASSO regression, random forest, and SVM-RFE. Genes consistently identified across all three methods were visualized with a Venn diagram and selected as candidate diagnostic markers. Functional similarity among the common DEGs was assessed using the “GOSemSim” R package by comparing them to reference gene sets, and the geometric mean of GO semantic similarity scores was calculated. Results were depicted with cloud and rain plots generated via ggplot2.

### Construction and validation of a risk score based on shared molecular features between UC and sarcopenia

Expression levels of the candidate diagnostic genes were examined in the UC and sarcopenia cohorts. Diagnostic performance was evaluated using receiver operating characteristic (ROC) analysis, and the area under the curve (AUC) was calculated. A multivariable logistic regression model was constructed in the training UC cohort, consisting of the merged GSE87466 and GSE75214 datasets. A nomogram was constructed using the “rms” R package to facilitate clinical interpretation, and model performance was assessed through ROC curves and AUC values. Calibration was evaluated via calibration plots and the concordance index (C-index), and 10-fold cross-validation was performed within the training cohort to assess model discrimination and reduce overfitting. The independent dataset GSE206285 was used exclusively for external validation.

### Immune infiltration landscape in UC and sarcopenia

Immune infiltration in UC and sarcopenia datasets was analyzed using the “ssGSEA” algorithm, quantifying the relative abundance of 28 immune cell subsets and comparing differences between groups. Associations between candidate diagnostic genes and immune cell levels were assessed using Spearman’s rank correlation. Hierarchical clustering heatmaps were generated with the “pheatmap” R package to illustrate the relationships between the two conserved genes and immune cell composition.

### Collection of clinical samples

Clinical records and paraffin-embedded colonic biopsy samples from hospitalized individuals with active UC and healthy controls were retrospectively reviewed at the Department of Gastroenterology, Renmin Hospital of Wuhan University. UC was diagnosed using integrated clinical, endoscopic, and histological criteria, and the study was approved by the institutional ethics committee (WDRY 2024-K114) with written informed consent obtained from all participants.

### Immunohistochemical staining

Paraffin sections were deparaffinized in xylene and rehydrated through graded ethanol. Antigen unmasking was performed by high-pressure heat treatment, followed by quenching of endogenous peroxidase activity using 3% hydrogen peroxide. After blocking with 10% goat serum, slides were incubated with primary antibodies at 4 °C overnight. Sections were then washed and exposed to secondary antibodies at room temperature for 1 h. Immunoreactivity was visualized using DAB, and nuclei were counterstained with hematoxylin. Images were captured with an upright microscope. Three randomly chosen high-power fields (400×) per section were analyzed, and average optical density values were quantified using Image J.

### Cell culture

NCM460 cells were maintained in Dulbecco’s modified Eagle medium containing 10% fetal bovine serum under standard incubator conditions (37 °C, 5% CO_2_). The culture medium was refreshed every two days, and subculturing was performed using 0.25% trypsin–EDTA when cell confluence approached 90%.

### Oil Red O staining

Cells were plated into 24-well culture dishes and maintained under standard conditions. Following an initial incubation period of 24 h, the culture medium was refreshed (500 μL per well). Cells were then exposed to lipopolysaccharide (1 μg/mL) for an additional 24 h. Subsequently, samples were rinsed with phosphate-buffered saline, chemically fixed using 4% paraformaldehyde, and stained with Oil Red O solution under light-protected conditions. Excess dye was eliminated through multiple washes with deionized water. Intracellular lipid deposition was evaluated by microscopic imaging and subsequent image-based analysis.

### Western blotting

Cells were lysed on ice with RIPA buffer, and supernatants were collected for protein extraction. Ten micrograms of protein were separated by 10% SDS-PAGE, transferred to PVDF membranes, and probed with PHYH primary antibody (Proteintech, 12858-1-AP) followed by secondary antibody incubation. Bands were visualized using an HRP substrate and imaged with a Bio-Rad system, and band intensities were quantified with ImageJ.

### Statistical analysis

Data analysis and visualization were performed in R (v4.2.3). Group comparisons used two-tailed Wilcoxon tests, correlations were assessed by Spearman’s method, and *P* < 0.05 was considered significant.

## Results

### Identification and enrichment analysis of shared DEGs in UC and sarcopenia

**Figure 2:**
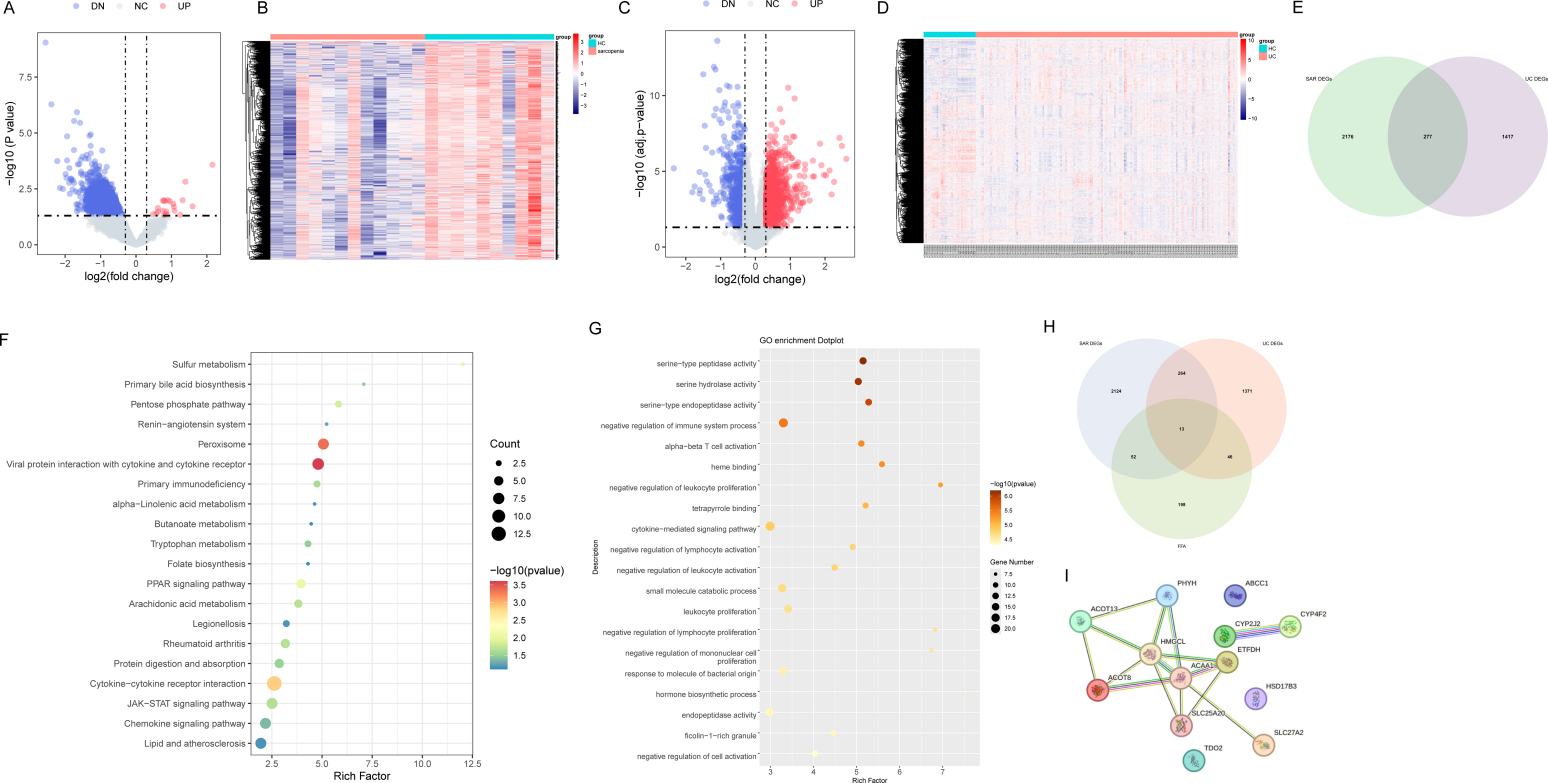
Identification of DEGs and the common genes in UC and sarcopenia. **(A)** Volcano plots showed DEGs of sarcopenia. **(B)** The DEG heatmap in the sarcopenia group. **(C)** The volcano plot of DEGs in UC patients. **(D)** The DEG heatmap in the UC group. **(E)** The shared DEGs between UC and sarcopenia by overlapping their DEGs. **(F)** Results of KEGG enrichment analysis of shared DEGs. **(G)** Results of GO enrichment analysis of shared DEGs. **(H)** Venn diagram: the intersection of DEGs in UC patients, DEGs in sarcopenia patients, and fatty acid metabolism-related genes. **(I)** The protein-protein network of fatty acid metabolism-related shared DEGs. HC, Healthy controls; UC, Ulcerative colitis. DEGs, differentially expressed genes.

Analysis of the sarcopenia dataset identified 2,453 DEGs (**Figures 2A, B**). To obtain UC-related DEGs, GSE87466 and GSE75214 were merged after batch effect correction, resulting in 1,694 DEGs (**Figures 2C, D**). Intersection of these two DEG sets yielded 277 shared DEGs (sDEGs), highlighting potential common molecular mechanisms between UC and sarcopenia (**Figure 2E**). KEGG pathway enrichment revealed that sDEGs were involved in key biological processes, including sulfur metabolism, the pentose phosphate pathway, α-linolenic acid metabolism, arachidonic acid metabolism, and lipid-associated atherosclerosis (**Figure 2F**). GO analysis indicated significant enrichment in serine-type peptidase and hydrolase activities, as well as negative regulation of immune processes (**Figure 2G**). Cross-referencing sDEGs with fatty acid metabolism-related genes identified 13 FAM-sDEGs (**Figure 2H**). A protein-protein interaction network was subsequently constructed using the STRING database to explore potential functional relationships (**Figure 2I**).

### Identification of fatty acid metabolism-related subtypes in UC

**Figure 3:**
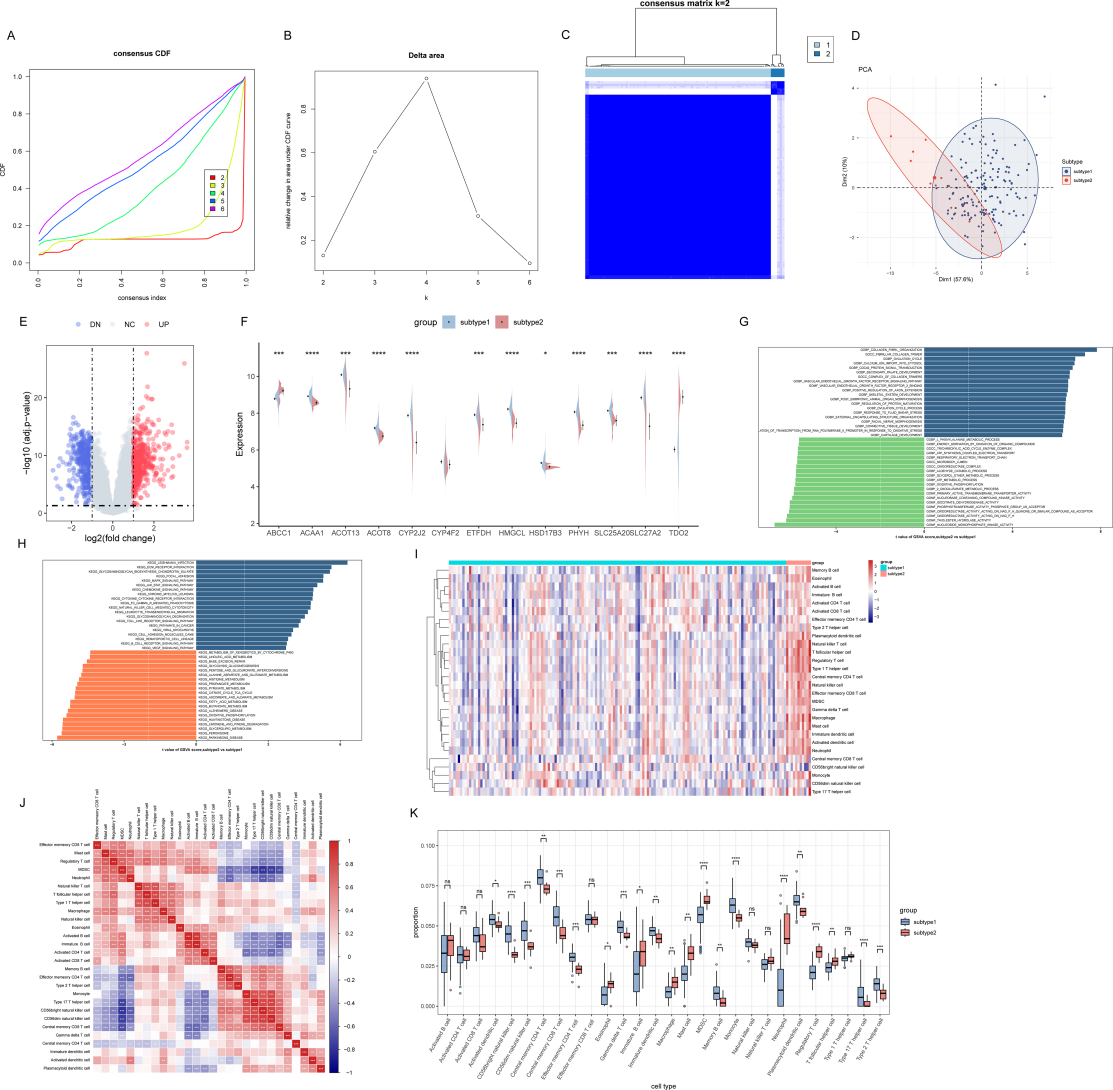
Identification of fatty acid metabolism-related UC subtypes. **(A)** Consistency CDF curves for unsupervised clustering when the number of clusters “k” is taken as 2–6. **(B)** Relative alterations in CDF delta area curves. **(C)** Consensus matrix heatmap when k=2. **(D)** Principal component analysis plot. **(E)** Gene expression volcano plot. **(F)** Violin plot comparing the expression levels of FAM-sDEGs between subtypes 1 and 2. **(G)** Biological functions analysis of UC subtypes ranked by *t* values of GSVA scores. **(H)** Hallmark pathways analysis of UC subtypes ranked by *t* values of GSVA scores. **(I)** The heatmap showed the distribution of different immune cell types in subtype 1 and subtype 2. **(J)** The heatmaps showed the correlation of immune cell infiltration. **(K)** Boxplot comparing the infiltration proportions of 28 types of immune cells between subtypes.

Unsupervised clustering of 161 patients with UC was performed using the 13 FAM-sDEGs. Optimal cluster separation was achieved at k = 2, as indicated by the consensus CDF plot (**Figure 3A**) and the delta area under the CDF curve (**Figure 3B**). Cluster stability was further supported by the consensus matrix heatmap, principal component analysis, and gene expression volcano plot (**Figures 3C–E**). Based on this classification, patients were assigned to two subtypes: subtype 1 (n = 150) and subtype 2 (n = 11). At the gene expression level, ABCC1 and TDO2 were markedly upregulated in subtype 2, whereas ACAA1, ACOT13, ACOT8, CYP2J2, ETFDH, HMGCL, HSD17B3, PHYH, SLC25A20, and SLC27A2 were significantly downregulated compared with subtype 1 (**Figure 3F**). Subtype 1 was thus characterized by elevated fatty acid metabolic activity.

To explore subtype-specific functional differences, GSVA was applied. In subtype 1, activities related to nucleoside monophosphate kinase, thiolester hydrolase, NADPH-dependent oxidoreductases, α-ketoglutarate metabolism, and ATP metabolic processes were significantly enriched, whereas subtype 2 showed enrichment in collagen fibril organization, fibrillar collagen trimer formation, and processes associated with the ovulatory cycle (**Figure 3G**). Subsequent KEGG pathway analysis indicated upregulation of peroxisome function, glycerolipid metabolism, limonene and pinene catabolism, oxidative phosphorylation, butanoate metabolism, and fatty acid metabolism in subtype 1, while extracellular matrix receptor interactions, chondroitin sulfate biosynthesis, focal adhesion, and MAPK signaling were elevated in subtype 2 (**Figure 3H**).

### Differences in immune cell infiltration between the subtypes

As shown in **Figures 3I, J**, among the 28 immune cell types evaluated by ssGSEA, substantial differences in immune cell infiltration quantities were observed between subtypes 1 and 2. The infiltration proportions of eosinophil, immature B cell, macrophage, mast cell, MDSC, neutrophil, regulatory T cell, and T follicular helper cell were significantly higher in subtype 2 than in subtype 1. In contrast, the infiltration proportions of activated dendritic cell, CD56 bright natural killer cell, CD56dim natural killer cell, central memory CD4 T cell, central memory CD8 T cell, effector memory CD4 T cell, gamma delta T cell, immature dendritic cell, memory B cell, monocyte, plasmacytoid dendritic cell, type 17 T helper cell, and type 2 T helper cell were significantly lower in subtype 2 than in subtype 1 (**Figure 3K**).

### Identification of hub FAM-sDEGs through machine learning

**Figure 4:**
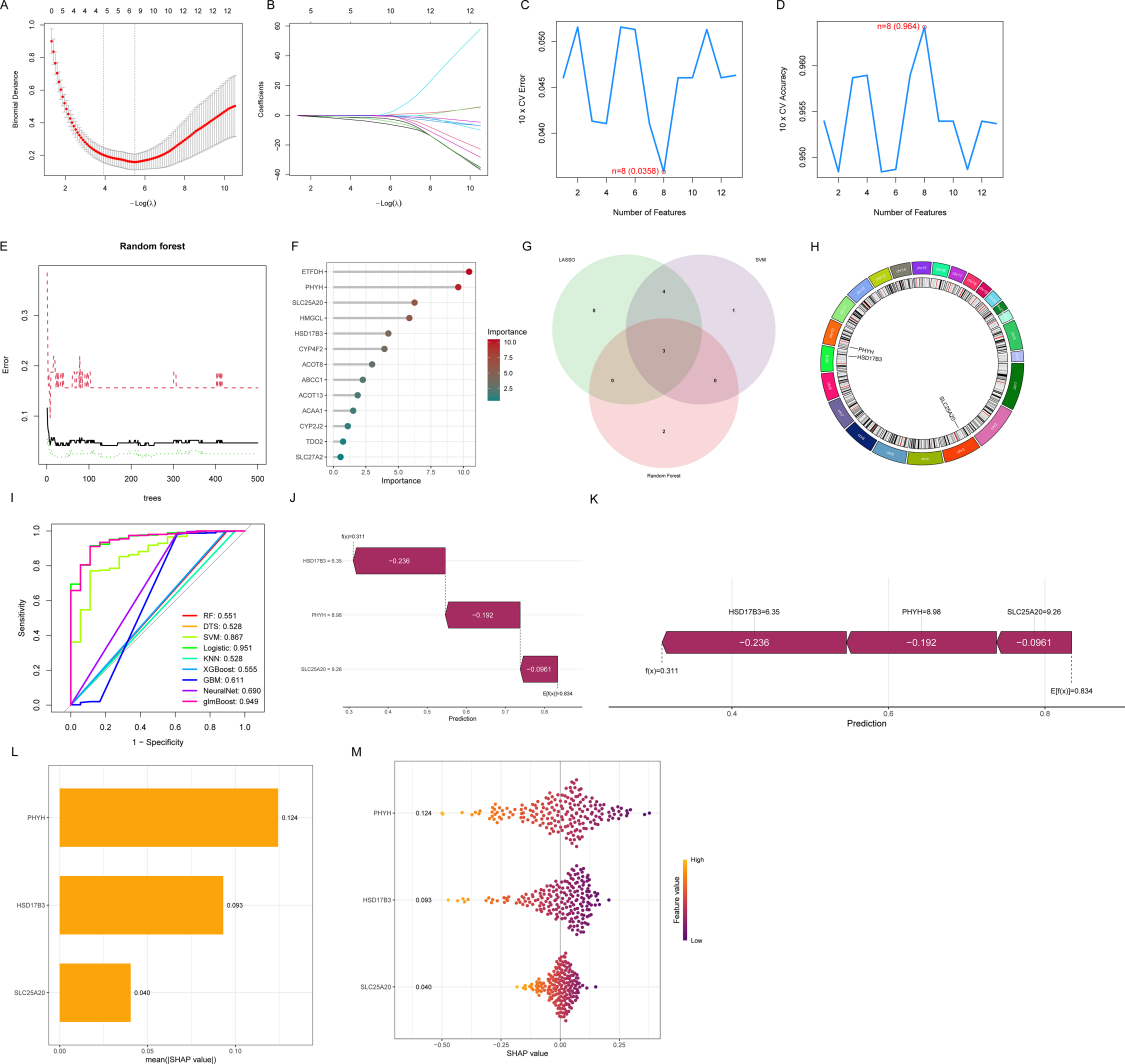
Screening of diagnostic genes by machine learning. **(A)** Path diagram of the LASSO regression model. **(B)** The optimal tuning parameter selection map of the LASSO algorithm. **(C)** SVM-RFE 10-fold cross-validation error rate. **(D)** SVM-RFE 10-fold cross-validation accuracy. **(E)** Random forest: relationship between tree number and error rate. **(F)** Top-10 genes according to their discriminant ability in the RF algorithm. **(G)** The Venn diagram showed three candidate diagnostic genes in UC by intersecting the results of LASSO, SVM-REF, and RF algorithms. **(H)** Chromosome localization of hub genes. **(I)** Nine machine learning algorithms were constructed to assess the hub genes. **(J, K)** Waterfall **(J)** and force plots **(K)** illustrated how individual samples were classified based on integrated predictions from hub genes. **(L)** Barplots presented the contribution magnitudes of the three genes in the logistic model. **(M)** The beeswarm plot.

Hub FAM-sDEGs were identified using three machine learning approaches. LASSO analysis selected seven feature genes from the training dataset (**Figures 4A, B**), while SVM-RFE identified eight characteristic genes (**Figures 4C, D**). In the random forest analysis, the top five genes ranked by importance were extracted (**Figures 4E, F**). Integration of these results highlighted three hub FAM-sDEGs: PHYH, HSD17B3, and SLC25A20 (**Figure 4G**). Chromosomal mapping revealed that PHYH is located on chromosome 10, HSD17B3 on chromosome 9, and SLC25A20 on chromosome 3 (**Figure 4H**).

### SHAP explanatory analysis

To validate the identified hub FAM-sDEGs, nine machine learning models were applied, including random forest, decision tree, logistic regression, K-nearest neighbors, XGBoost, gradient boosting, neural network, and glmBoost (**Figure 4I**). Logistic regression demonstrated the highest overall predictive accuracy, with the greatest AUC. To interpret this model, SHAP values were computed using the “shapviz” R package, and genes were ranked based on their contribution. Waterfall and force plots quantified the cumulative influence of PHYH, HSD17B3, and SLC25A20 on individual sample predictions, providing insight into sample-level classification (**Figures 4J, K**). Barplot analysis identified PHYH as the strongest contributor, while beewarm plots emphasized its central role in classification, confirming that all three genes exerted positive effects on the model output (**Figures 4L, M**). These results indicate that reduced expression of these genes is associated with the disease phenotype compared to controls.

### Development and validation of the LR model

**Figure 5:**
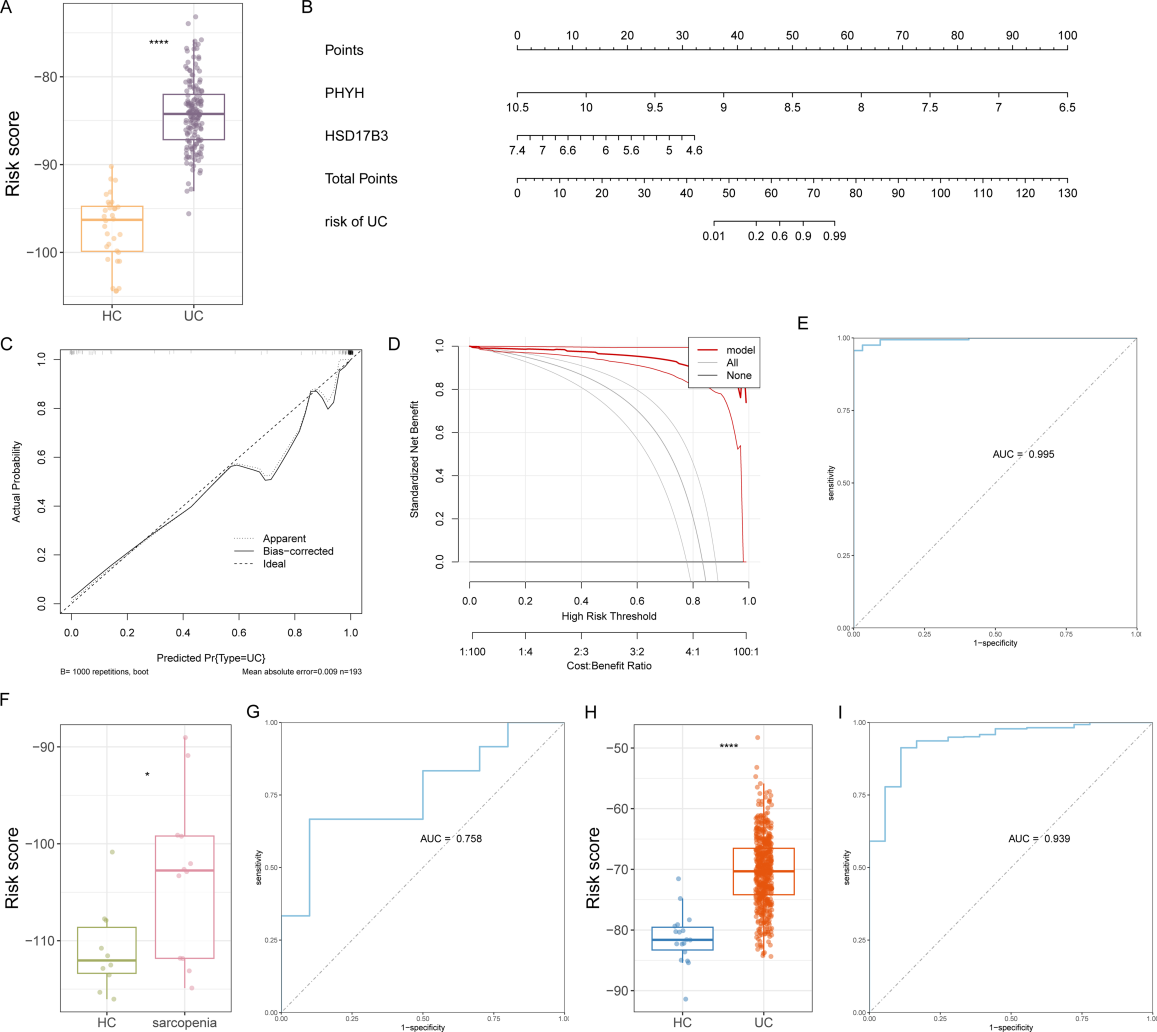
Construction of predictive scoring model. **(A)** Logistic model for the prediction of UC risk. **(B)** Nomogram predicting the probability of UC. **(C-E)** Calibration curves **(C)**, DCA **(D)**, and ROC **(E)** evaluating the prediction efficacy and clinical benefit of the nomogram in the training set. **(F)** The risk scores calculated by the model for patients with sarcopenia and HC. **(G)** ROC curve risk model for sarcopenia. **(H)** The risk scores calculated by the model for patients with UC and HC in the validation set GSE206285. **(I)** ROC curve risk model in the validation set GSE206285.

A logistic regression model was developed based on the hub FAM-sDEGs, with the risk score defined as (-8.0620 × PHYH) + (-3.7081 × HSD17B3). In the training cohort, patients with UC exhibited significantly higher risk scores than healthy controls (**Figure 5A**). To facilitate clinical interpretation, a nomogram was constructed, and calibration and decision curve analyses confirmed the model’s strong predictive performance for UC, with an AUC of 0.995 (**Figures 5B–E**). Similarly, sarcopenia patients showed elevated risk scores relative to controls, and the model achieved an AUC of 0.758 in this cohort, outperforming individual hub genes (**Figures 5F, G**).

For external validation, the GSE206285 dataset was analyzed, showing that UC patients had markedly higher risk scores than healthy controls (**Figure 5H**). The model achieved an AUC of 0.939 in this cohort, outperforming predictions based on individual hub genes (**Figure 5I**). These findings highlight the improved discriminative ability of the multi-gene model and support its potential for broader clinical application.

### Diagnostic efficacy of hub genes

**Figure 6:**
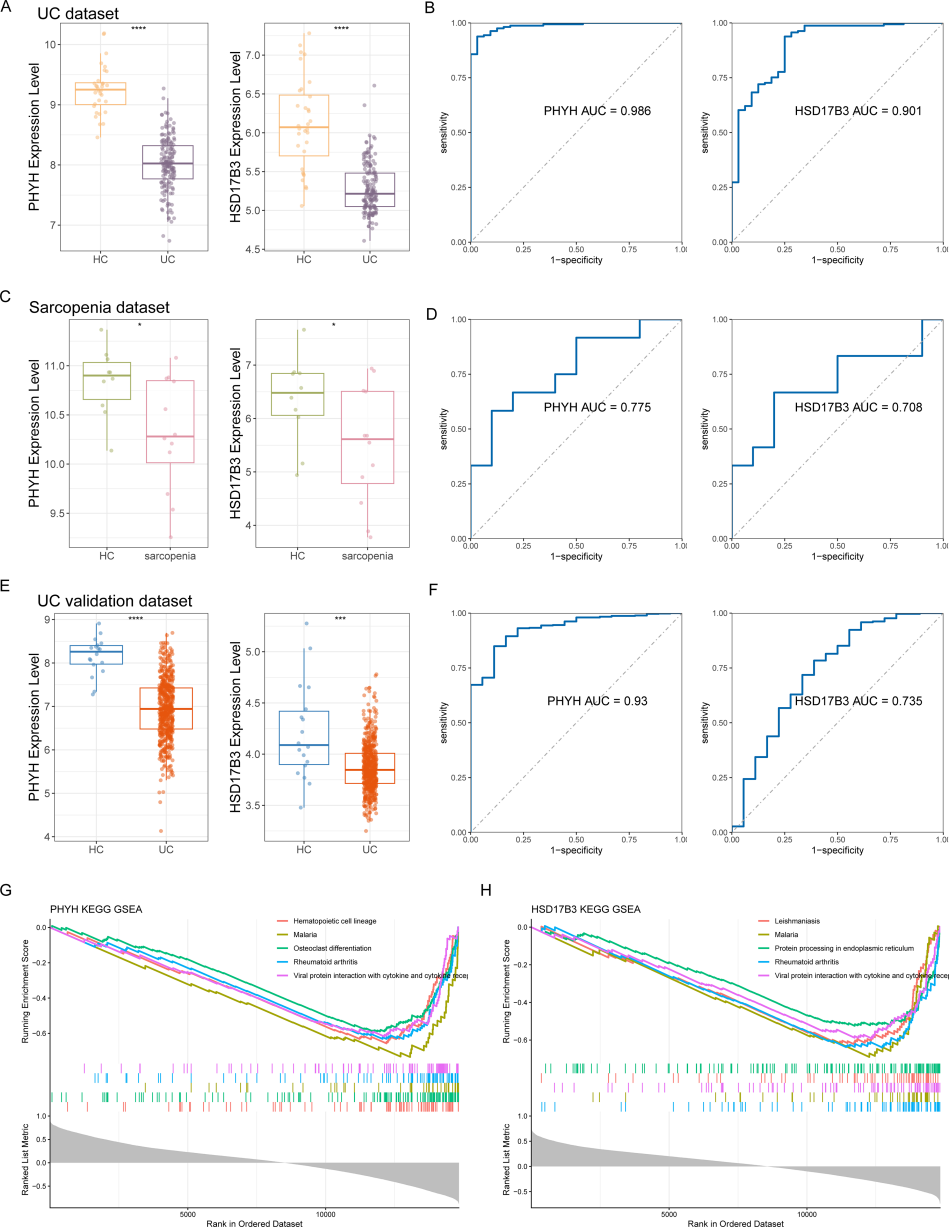
**(A)** The expression level of the feature genes in UC and HC. **(B)** ROC curves of PHYH and HSD17B3 for UC. **(C)** The expression level of the feature genes in sarcopenia and HC. **(D)** ROC curves of PHYH and HSD17B3 for sarcopenia. **(E)** The expression level of the feature genes in the external dataset GSE206285 for UC. **(F)** ROC curves of PHYH and HSD17B3 in the external dataset GSE206285 for UC. **(G)** GSEA of PHYH. **(H)** GSEA of HSD17B3.

Single-gene expression analyses were performed for the three candidate biomarkers. In the UC cohort, PHYH and HSD17B3 exhibited significant downregulation compared with healthy controls (**Figure 6A**), with AUC values of 0.986 and 0.901, respectively, indicating strong diagnostic performance (**Figure 6B**). In the sarcopenia dataset, both genes were decreased in patients relative to controls, with more modest AUCs of 0.775 for PHYH and 0.708 for HSD17B3 (**Figures 6C, D**), suggesting limited predictive power in this context. External validation using the GSE206285 dataset confirmed reduced expression of PHYH and HSD17B3 in UC samples (**Figure 6E**), with corresponding AUCs of 0.930 and 0.735 (**Figure 6F**), supporting their diagnostic relevance.

### GSEA of the hub genes

To investigate potential mechanisms of hub genes, single-gene GSEA was conducted. PHYH expression was enriched in pathways related to Th17 cell differentiation and cell adhesion molecules (**Figure 6G**), whereas HSD17B3 expression correlated positively with endoplasmic reticulum protein processing (**Figure 6H**). These results provide insights into the molecular functions of the hub genes and suggest potential targets for therapeutic intervention.

### Immune microenvironment of the patients with UC

**Figure 7:**
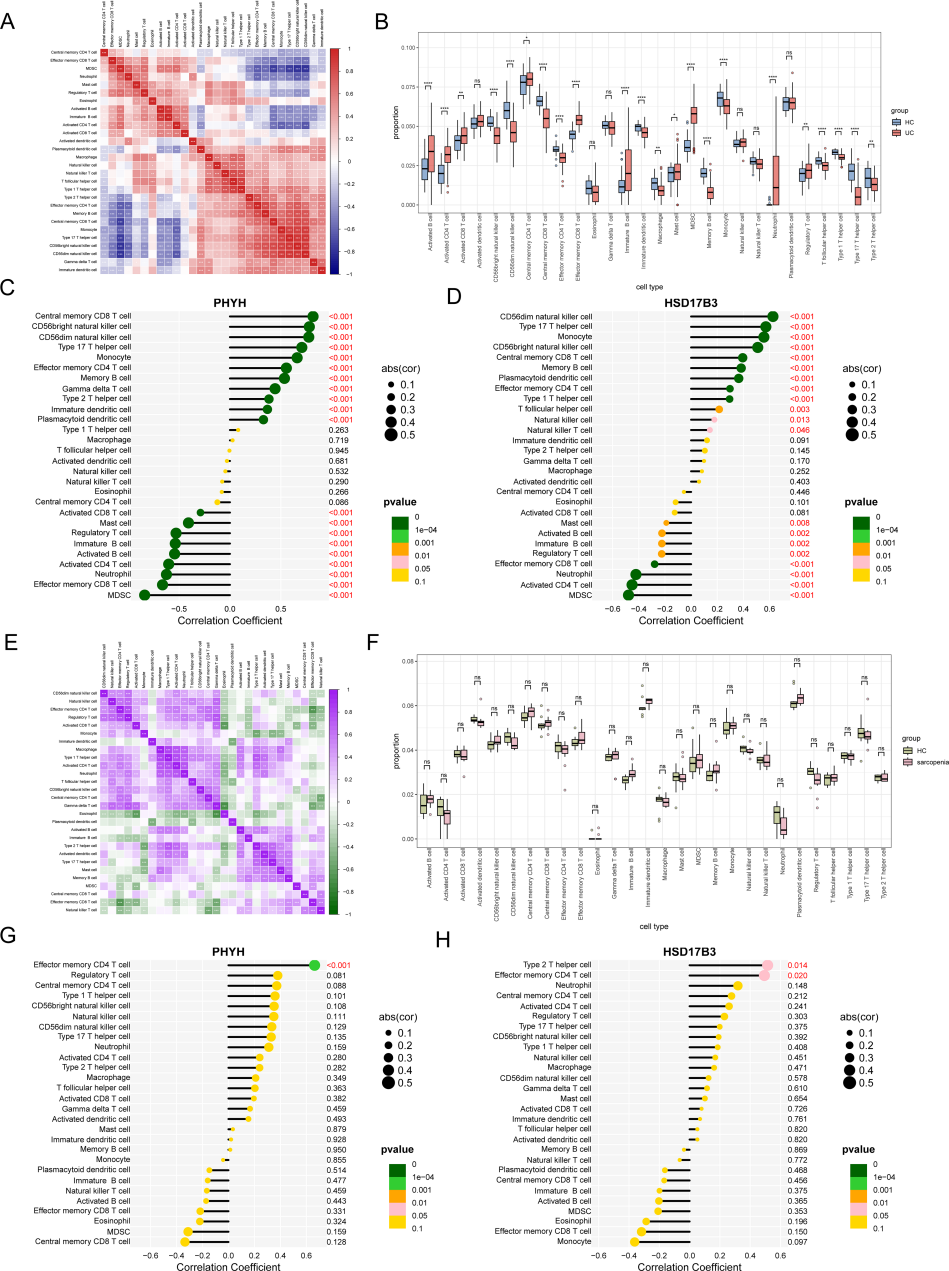
**(A)** The heatmaps showed the correlation of immune cell infiltration in UC and HC. **(B)** Boxplot comparing the infiltration proportions of 28 types of immune cells in UC and HC. **(C, D)** The lollipop plot showed the correlation coefficient of PHYH **(C)** and HSD17B3 **(D)** and immune cells in the dataset of UC. **(E)** The heatmaps showed the correlation of immune cell infiltration in sarcopenia and HC. **(F)** Boxplot comparing the infiltration proportions of 28 types of immune cells in sarcopenia and HC. **(G, H)** The lollipop plot showed the correlation coefficient of PHYH **(G)** and HSD17B3 **(H)** and immune cells in the dataset of sarcopenia.

To explore the dissimilarities in the immune microenvironment of patients with UC versus healthy controls, we initially analyzed the distribution patterns of 28 immune cell types (**Figure 7A**). Meanwhile, a detailed examination of the numerical differences in these immune cell populations between the two groups was carried out (**Figure 7B**). The results indicated that there were 28 immune cell populations showing significant differences in abundance in UC samples. In particular, compared with healthy controls, patients with UC had a higher infiltration of activated B cells, activated CD4 T cells, activated CD8 T cells, effector memory CD8 T cells, immature B cells, MDSC, and neutrophils. The correlation analysis between hub genes and distinct immune cell subsets revealed significant associations with a diverse range of immune cell populations (**Figures 7C, D**). These results provide crucial insights into the interplay between PHYH and HSD17B3 and the immune microenvironment in UC.

### Immune microenvironment of patients with sarcopenia

To explore the dissimilarities in the immune microenvironment of patients with sarcopenia versus healthy controls, we initially analyzed the distribution patterns of 28 immune cell types (**Figure 7E**). Meanwhile, a detailed examination of the numerical differences in these immune cell populations between the two groups was carried out (**Figure 7F**). The correlation analysis between hub sDEGs and distinct immune cell subsets revealed PHYH was strongly correlated with effector memory CD4 T cell, and HSD17B3 was strongly correlated with type 2 T helper cell and effector memory CD4 T cell (**Figures 7G, H**). These results provide crucial insights into the interplay between hub sDEGs and the immune microenvironment in sarcopenia.

### Preliminary validation of the fatty acid metabolism and hub genes

**Figure 8:**
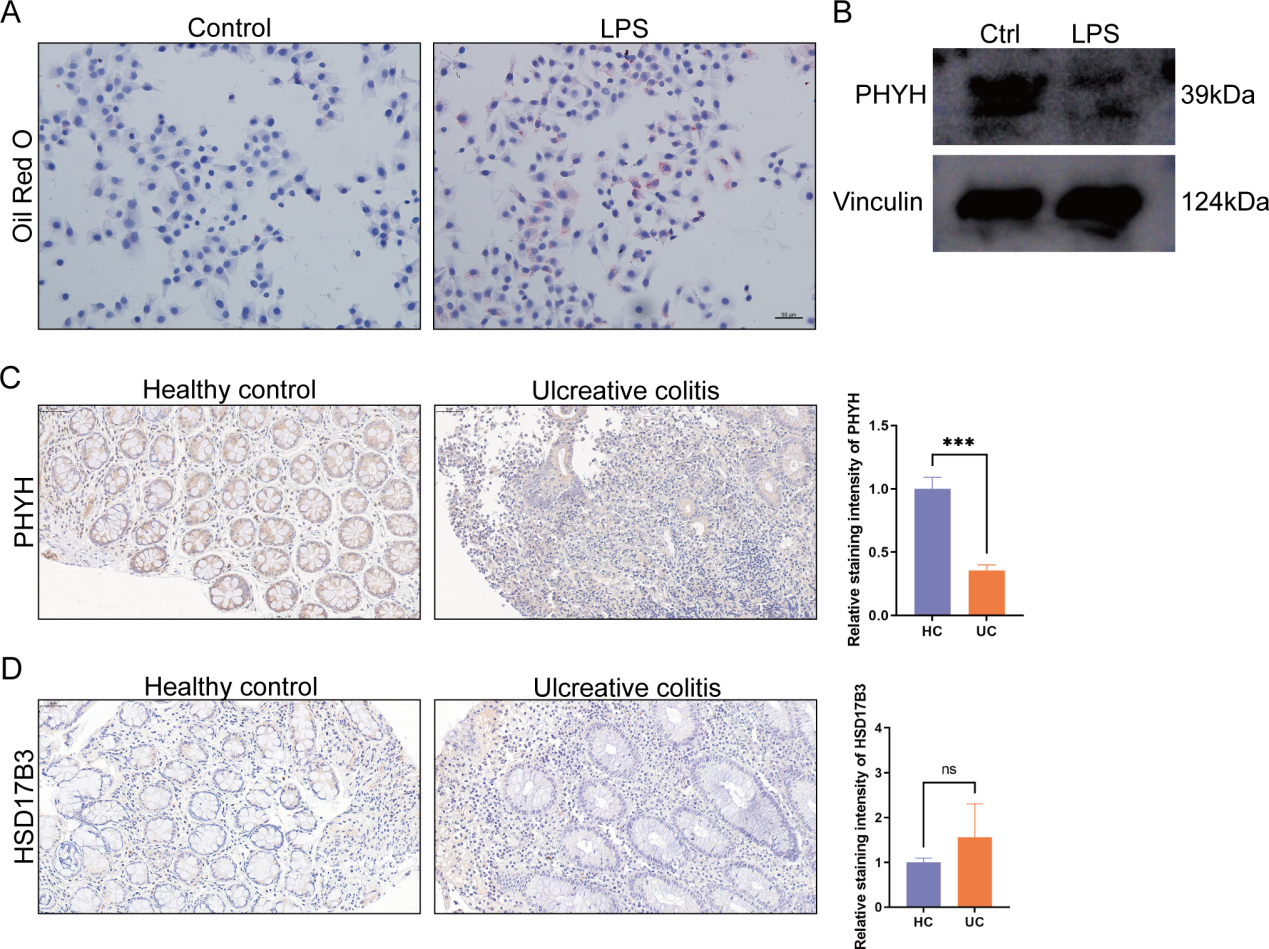
**(A)** Images of Oil Red O staining on NCM460 cells, as indicated (scale bars: 50 µm). **(B)** The expression of PHYH proteins in cells. Vinculin was used as a loading control. **(C)** Immunohistochemical staining of PHYH in colon sections of healthy controls and patients with UC. **(D)** Immunohistochemical staining of HSD17B3 in colon sections of healthy controls and patients with UC. (***<0.001, scale bar: 50 μm).

Oil Red O staining revealed abundant intracellular lipid droplets, indicating that LPS treatment induced dysregulation of fatty acid metabolism in NCM460 cells (**Figure 8A**). Western blot analysis showed that PHYH expression was reduced following LPS exposure (**Figure 8B**). Immunohistochemistry further confirmed that PHYH protein levels in colonic biopsy specimens from UC patients were significantly lower than in controls, consistent with the bioinformatic predictions (**Figure 8C**). However, immunohistochemistry revealed no significant difference in HSD17B3 expression between colonic biopsy specimens from UC patients and controls (**Figure 8D**). This discrepancy may be attributed to limited sample size, inter-individual variability, differences in detection sensitivity between transcriptomic and protein-level assays, or the influence of post-transcriptional regulation.

## Discussion

This study aimed to delineate transcriptomic features shared by UC and sarcopenia, and to further evaluate whether fatty acid metabolism contributes to UC heterogeneity and immune microenvironment differences. By integrating multiple UC cohorts with batch-effect correction and analyzing an independent sarcopenia dataset, we identified sDEGs, suggesting that the two conditions may converge on partially overlapping molecular programs. GO/KEGG enrichment of sDEGs highlighted not only immune-regulatory processes but also several metabolism-related pathways, including sulfur metabolism, the pentose phosphate pathway, and lipid-associated pathways. These findings support the view that chronic inflammation is accompanied by broad metabolic remodeling, which may provide an important mechanistic bridge between intestinal inflammation and systemic phenotypes (20).

Notably, lipid-related signals were prominent among enriched pathways, particularly those involving arachidonic acid and alpha-linolenic acid metabolism. Lipid mediators produced along these routes can influence epithelial barrier stability, leukocyte recruitment, and the duration and magnitude of mucosal inflammation, providing a plausible link to UC pathogenesis and its systemic metabolic consequences (21). In parallel, enrichment for serine-type peptidase/hydrolase activity suggests active proteolysis and tissue remodeling in disease tissue. Such processes may amplify inflammation through effects on antigen processing, extracellular matrix (ECM) turnover, and activation of downstream inflammatory signaling. Taken together, our results indicate that the overlap between UC and sarcopenia is not limited to immune activation but also involves coordinated changes in metabolic networks (5).

Building on these observations, we focused on fatty acid metabolism by intersecting sDEGs with curated fatty acid metabolism gene sets to derive 13 FAM-sDEGs, and used these genes to perform unsupervised clustering in the UC cohort. Two metabolism-associated subtypes emerged. Subtype 1 displayed an expression profile consistent with relatively higher fatty acid metabolic activity, whereas subtype 2 showed reduced expression of multiple fatty acid metabolism genes, with upregulation of a small subset such as ABCC1 and TDO2. This pattern suggests that UC patients are metabolically heterogeneous and that a subset may exhibit a suppressed fatty acid metabolism signature. Potential drivers include inflammatory signaling, local hypoxia, altered epithelial energy utilization, or compositional shifts in immune and stromal cells that influence bulk transcriptomes. Regardless of the underlying cause, this classification provided a useful framework for interpreting downstream pathway activity and immune infiltration differences (22–24).

GSVA and pathway enrichment analyses further supported functional divergence between subtypes. Subtype 1 was enriched for redox metabolism, ATP metabolism, oxidative phosphorylation, peroxisome-related pathways, and fatty acid metabolism, indicating stronger bioenergetic capacity and lipid handling and implicating mitochondrial and peroxisomal functions. In contrast, subtype 2 was characterized by enrichment of ECM receptor interaction, focal adhesion, glycosaminoglycan biosynthesis, and MAPK signaling, suggesting a tissue state with enhanced structural remodeling and cell–matrix communication. Given the close links between ECM remodeling and immune cell migration, adhesion, and persistence, subtype 2 may represent a microenvironment more permissive to continuous immune cell recruitment and chronic inflammation (25).

Immune infiltration analysis was consistent with this interpretation. Subtype 2 exhibited higher infiltration of neutrophils, macrophages, MDSCs, mast cells, eosinophils, and regulatory T cells, while NK cells and several memory T-cell and dendritic-cell–related populations were comparatively reduced. This profile appears more myeloid-dominant, with features of immunoregulatory feedback and tissue remodeling. Increased neutrophils and macrophages typically reflect heightened inflammatory activity, whereas elevated MDSCs and Tregs may indicate compensatory immunosuppression in a chronic inflammatory context. Reduced NK and certain memory T-cell subsets may arise from altered trafficking, local exhaustion, or microenvironmental constraints. Overall, these results suggest that fatty acid metabolism–based stratification captures not only metabolic variation but also distinct immune organizational states, underscoring potential immune–metabolic coupling in UC (26, 27).

To identify stable and clinically relevant biomarkers, we applied multiple machine-learning approaches (LASSO, SVM-RFE, and random forest) and converged on three hub genes: PHYH, HSD17B3, and SLC25A20. All three were consistently downregulated in both UC and sarcopenia, supporting their relevance to shared molecular changes. Functionally, PHYH encodes a peroxisomal enzyme involved in alpha-oxidation, and SLC25A20 encodes the carnitine–acylcarnitine translocase required for mitochondrial fatty acid beta-oxidation. Their downregulation is therefore compatible with reduced fatty acid oxidation capacity. HSD17B3 is classically associated with steroid metabolism, but its downregulation here may also reflect broader metabolic reprogramming. SHAP-based interpretability analyses further indicated that PHYH contributed most strongly to classification, suggesting that it may represent a key metabolic node in the model.

In diagnostic assessments, PHYH and HSD17B3 showed strong discriminatory performance in the UC training cohort and remained informative in an external validation cohort, indicating reasonable robustness. In sarcopenia, single-gene AUC values were modest, which may reflect the clinical and biological heterogeneity of sarcopenia, variability in tissue composition and disease stage, and dilution of transcriptomic signals by comorbidities (28, 29). Importantly, a logistic regression risk model integrating hub genes achieved superior performance relative to single genes and showed improved generalizability in sarcopenia-related samples, highlighting the advantage of multi-gene integration for capturing complex disease states.

Single-gene GSEA offered additional mechanistic clues. PHYH expression was associated with Th17 cell differentiation and cell adhesion molecule pathways, suggesting that peroxisomal lipid metabolism may relate to immune polarization and cell trafficking/adhesion programs. HSD17B3 expression was linked to protein processing in the endoplasmic reticulum, implicating stress responses and ER homeostasis, which are known to contribute to epithelial dysfunction and inflammatory amplification. While these associations are not causal, they provide testable hypotheses whereby reduced fatty acid handling capacity might promote metabolic and proteostasis stress and reshape immune networks, thereby sustaining inflammation.

We also observed broad immune cell shifts between UC and healthy controls, including higher levels of activated B/T cells, neutrophils, and MDSCs, consistent with the inflammatory and myeloid expansion features of UC. Correlations between hub genes and immune subsets suggest that PHYH and HSD17B3 may serve as molecular readouts linking metabolic remodeling to immune microenvironment changes (30, 31). In sarcopenia, associations between hub genes and specific T-cell subsets similarly suggest that immune alterations may accompany metabolic signatures, though the precise patterns likely depend on tissue context and clinical phenotype.

From a translational perspective, the fatty acid metabolism–related subtypes proposed here provide a new angle for interpreting UC heterogeneity. Patients with distinct metabolic states may exhibit different immune infiltration patterns and pathway activities, which could influence therapeutic response and prognosis (32). Moreover, the external validation of PHYH and HSD17B3 and the performance of the integrated risk model support the feasibility of developing simplified biomarker panels or risk assessment tools.

Despite these findings, several limitations should be acknowledged. First, the public datasets lacked comprehensive clinical annotation, and potential confounders such as disease duration, disease severity, treatment history, and nutritional status could not be uniformly controlled. In particular, the sarcopenia-related analysis relied solely on the GSE1428 cohort, which had a relatively small sample size (n = 22); therefore, these results should be interpreted with caution and further validated in larger, independent cohorts. Second, bulk transcriptomic data cannot fully distinguish cell-intrinsic regulatory alterations from changes in cellular composition, and the ssGSEA-based immune infiltration analysis remains a computational inference that requires further confirmation by single-cell or spatial transcriptomics as well as experimental approaches. Finally, although preliminary experimental validation was performed, protein-level validation of HSD17B3 and functional studies remain insufficient. Further mechanistic investigations are needed to clarify the specific roles of these hub genes in lipid metabolism, immune regulation, and inflammation.

In summary, our study reveals shared immune–metabolic transcriptomic features between UC and sarcopenia and suggests that fatty acid metabolism is a key factor shared with UC molecular heterogeneity and immune infiltration differences. The hub genes PHYH and HSD17B3 showed consistent signals across machine-learning selection, interpretability analyses, and external validation, and the integrated risk model further demonstrated the benefit of combining complementary metabolic signals. Together, these findings provide a set of testable candidates and a potential framework for future mechanistic and translational studies.

## Data Availability

The datasets presented in this study can be found in online repositories. The names of the repository/repositories and accession number(s) can be found in the article/**Supplementary Material**.

## References

[1] Wangchuk P Yeshi K Loukas A . Ulcerative colitis: clinical biomarkers, therapeutic targets, and emerging treatments. Trends Pharmacol Sci. (2024) 45:892–903. doi: 10.1016/j.tips.2024.08.003. PMID: 39261229

[2] Liang Y Li Y Lee C Yu Z Chen C Liang C . Ulcerative colitis: molecular insights and intervention therapy. Mol BioMed. (2024) 5:42. doi: 10.1186/s43556-024-00207-w. PMID: 39384730 PMC11464740

[3] Le Berre C Honap S Peyrin-Biroulet L . Ulcerative colitis. Lancet. (2023) 402:571–84. doi: 10.1016/S0140-6736(23)00966-2. PMID: 37573077

[4] Yuan S Larsson SC . Epidemiology of sarcopenia: Prevalence, risk factors, and consequences. Metabolism. (2023) 144:155533. doi: 10.1016/j.metabol.2023.155533. PMID: 36907247

[5] Zhang Y Zhang L Gao X Dai C Huang Y Wu Y . Impact of malnutrition and sarcopenia on quality of life in patients with inflammatory bowel disease: A multicentre study. J Cachexia Sarcopenia Muscle. (2023) 14:2663–75. doi: 10.1002/jcsm.13341. PMID: 37779327 PMC10751433

[6] Neelam PB Sharma A Sharma V . Sarcopenia and frailty in inflammatory bowel disease: Emerging concepts and evidence. JGH Open. (2024) 8:e13033. doi: 10.1002/jgh3.13033. PMID: 38283070 PMC10821747

[7] Nishikawa H Nakamura S Miyazaki T Kakimoto K Fukunishi S Asai A . Inflammatory bowel disease and sarcopenia: Its mechanism and clinical importance. J Clin Med. (2021) 10:4214. doi: 10.3390/jcm10184214. PMID: 34575326 PMC8470813

[8] Li X . Sarcopenia and acute severe ulcerative colitis patients. Dig Liver Dis. (2021) 53:1521. doi: 10.1016/j.dld.2021.05.010. PMID: 34103234

[9] Neelam PB Pal R Gupta P Singh AK Shah J Mandavdhare HS . Sarcopenia is common in ulcerative colitis and correlates with disease activity. Intest Res. (2024) 22:162–71. doi: 10.5217/ir.2023.00090. PMID: 38247117 PMC11079510

[10] Millan-Domingo F Garcia-Dominguez E Gambini J Olaso-Gonzalez G Viña J Gomez-Cabrera MC . Diet and exercise in frailty and sarcopenia. Molecular aspects. Mol Aspects Med. (2024) 100:101322. doi: 10.1016/j.mam.2024.101322. PMID: 39591800

[11] Sun S Xu X Liang L Wang X Bai X Zhu L . Lactic acid-producing probiotic Saccharomyces cerevisiae attenuates ulcerative colitis via suppressing macrophage pyroptosis and modulating gut microbiota. Front Immunol. (2021) 12:777665. doi: 10.3389/fimmu.2021.777665. PMID: 34899735 PMC8652295

[12] Ye Z Deng M Yang Y Song Y Weng L Qi W . Epithelial mitochondrial fission-mediated PANoptosis is crucial for ulcerative colitis and its inhibition by saquinavir through Drp1. Pharmacol Res. (2024) 210:107538. doi: 10.1016/j.phrs.2024.107538. PMID: 39643069

[13] Nie Y Meng W Liu D Yang Z Wang W Ren H . Exosomes derived from apical papilla stem cells improve NASH by regulating fatty acid metabolism and reducing inflammation. Mol Med. (2024) 30:186. doi: 10.1186/s10020-024-00945-1. PMID: 39462343 PMC11512503

[14] Wang H Zhu W Hong Y Wei W Zheng N He X . Astragalus polysaccharides attenuate chemotherapy-induced immune injury by modulating gut microbiota and polyunsaturated fatty acid metabolism. Phytomedicine. (2024) 128:155492. doi: 10.1016/j.phymed.2024.155492. PMID: 38479258

[15] Deng B Liu Y Chen Y He P Ma J Tan Z . Exploring the butyrate metabolism-related shared genes in metabolic associated steatohepatitis and ulcerative colitis. Sci Rep. (2024) 14:15949. doi: 10.1038/s41598-024-66574-0. PMID: 38987612 PMC11237055

[16] Deng B Zhen J Xiang Z Li X Tan C Chen Y . Unveiling and validating the role of fatty acid metabolism in ulcerative colitis. J Inflammation Res. (2024) 17:6345–62. doi: 10.2147/JIR.S479011. PMID: 39291081 PMC11407323

[17] Zhang Y Ji W Qin H Chen Z Zhou Y Zhou Z . Astragalus polysaccharides alleviate DSS-induced ulcerative colitis in mice by restoring SCFA production and regulating Th17/Treg cell homeostasis in a microbiota-dependent manner. Carbohydr Polym. (2025) 349:122829. doi: 10.1016/j.carbpol.2024.122829. PMID: 39643403

[18] Therdyothin A Phiphopthatsanee N Isanejad M . The effect of omega-3 fatty acids on sarcopenia: Mechanism of action and potential efficacy. Mar Drugs. (2023) 21:399. doi: 10.3390/md21070399. PMID: 37504930 PMC10381755

[19] Al Saedi A Debruin DA Hayes A Hamrick M . Lipid metabolism in sarcopenia. Bone. (2022) 164:116539. doi: 10.1016/j.bone.2022.116539. PMID: 36007811

[20] Kinchen J Chen HH Parikh K Antanaviciute A Jagielowicz M Fawkner-Corbett D . Structural remodeling of the human colonic mesenchyme in inflammatory bowel disease. Cell. (2018) 175:372–386.e17. doi: 10.1016/j.cell.2018.08.067. PMID: 30270042 PMC6176871

[21] Stephens M von der Weid P-Y . Lipopolysaccharides modulate intestinal epithelial permeability and inflammation in a species-specific manner. Gut Microbes. (2020) 11:421–32. doi: 10.1080/19490976.2019.1629235. PMID: 31203717 PMC7524286

[22] Vassiliou E Farias-Pereira R . Impact of lipid metabolism on macrophage polarization: Implications for inflammation and tumor immunity. Int J Mol Sci. (2023) 24:12032. doi: 10.3390/ijms241512032. PMID: 37569407 PMC10418847

[23] He J Zhang P Shen L Niu L Tan Y Chen L . Short-chain fatty acids and their association with signalling pathways in inflammation, glucose and lipid metabolism. Int J Mol Sci. (2020) 21:6356. doi: 10.3390/ijms21176356. PMID: 32887215 PMC7503625

[24] Luo Z Chen Z Hu J Ding G . Interplay of lipid metabolism and inflammation in podocyte injury. Metabolism. (2024) 150:155718. doi: 10.1016/j.metabol.2023.155718. PMID: 37925142

[25] Chen C Chen J Wang Y Fang L Guo C Sang T . Ganoderma lucidum polysaccharide inhibits HSC activation and liver fibrosis via targeting inflammation, apoptosis, cell cycle, and ECM-receptor interaction mediated by TGF-β/Smad signaling. Phytomedicine. (2023) 110:154626. doi: 10.1016/j.phymed.2022.154626. PMID: 36603342

[26] Liang J Dai W Liu C Wen Y Chen C Xu Y . Gingerenone A attenuates ulcerative colitis via targeting IL-17RA to inhibit inflammation and restore intestinal barrier function. Adv Sci (Weinh). (2024) 11:e2400206. doi: 10.1002/advs.202400206. PMID: 38639442 PMC11267284

[27] Wu Y Zhang X Liu X Zhao Z Tao S Xu Q . Galactooligosaccharides and Limosilactobacillus reuteri synergistically alleviate gut inflammation and barrier dysfunction by enriching Bacteroides acidifaciens for pentadecanoic acid biosynthesis. Nat Commun. (2024) 15:9291. doi: 10.1038/s41467-024-53144-1. PMID: 39468026 PMC11519483

[28] Daich Varela M Schiff E Malka S Wright G Mahroo OA Webster AR . PHYH c.678+5G>T leads to in-frame exon skipping and is associated with attenuated Refsum disease. Invest Ophthalmol Vis Sci. (2024) 65:38. doi: 10.1167/iovs.65.2.38. PMID: 38411969 PMC10910431

[29] Lawrence BM O’Donnell L Smith LB Rebourcet D . New insights into testosterone biosynthesis: Novel observations from HSD17B3 deficient mice. Int J Mol Sci. (2022) 23:15555. doi: 10.3390/ijms232415555. PMID: 36555196 PMC9779265

[30] Adam M Heikelä H Sobolewski C Portius D Mäki-Jouppila J Mehmood A . Hydroxysteroid (17β) dehydrogenase 13 deficiency triggers hepatic steatosis and inflammation in mice. FASEB J. (2018) 32:3434–47. doi: 10.1096/fj.201700914R. PMID: 29401633

[31] Weng Z Zheng X Liang Y Chen X Peng Q Zhang G . Porcine alveolar macrophages host proteins interacting with African swine fever virus p72. Front Microbiol. (2024) 15:1370417. doi: 10.3389/fmicb.2024.1370417. PMID: 38481793 PMC10932996

[32] Netea MG Joosten LAB Latz E Mills KHG Natoli G Stunnenberg HG . Trained immunity: A program of innate immune memory in health and disease. Science. (2016) 352:aaf1098. doi: 10.1126/science.aaf1098. PMID: 27102489 PMC5087274

